# Targeting Protein Tyrosine Phosphatase 1B: Recent Advances in Natural, Synthetic, and Multitarget Inhibitors for Diabetes Therapy

**DOI:** 10.3390/biom16071058

**Published:** 2026-07-19

**Authors:** Laura Braconi, Lorenzo Mattolini, Maria Novella Romanelli, Elisabetta Teodori, Dina Manetti

**Affiliations:** Department of Neurosciences, Psychology, Drug Research and Child’s Health-Section of Pharmaceutical and Nutraceutical Sciences, Via U. Schiff 6, 50019 Sesto Fiorentino, Italy; laura.braconi@unifi.it (L.B.); lorenzo.mattolini@unifi.it (L.M.); novella.romanelli@unifi.it (M.N.R.); elisabetta.teodori@unifi.it (E.T.)

**Keywords:** protein tyrosine phosphatase 1B, PTP1B inhibitors, type 2 diabetes mellitus, Insulin resistance, allosteric inhibitors, multitarget drug design, natural products, LMW-PTP

## Abstract

Diabetes mellitus, particularly type 2 diabetes mellitus (T2DM), represents a major global health challenge, driven by the increasing prevalence of obesity and sedentary lifestyles. T2DM is characterized by insulin resistance and progressive β-cell dysfunction, leading to chronic hyperglycemia and multiple complications. Among the molecular targets investigated for therapeutic intervention, protein tyrosine phosphatase 1B (PTP1B) has emerged as a key negative regulator of insulin signaling. By dephosphorylating the insulin receptor and its downstream substrates, PTP1B attenuates insulin action and contributes to metabolic dysfunction. In addition to its role in glucose homeostasis, PTP1B is implicated in obesity, diabetic complications, neurodegenerative disorders, and cancer, highlighting its relevance as a multifunctional therapeutic target. However, the development of PTP1B inhibitors remains challenging due to the highly conserved and polar nature of its catalytic site, which limits selectivity and cell permeability. Recent research has focused on alternative strategies, including allosteric modulation and multi-site inhibition, to overcome these limitations. This review provides a comprehensive overview of PTP1B inhibitors from both synthetic (2019–2025) and natural sources, with particular emphasis on natural products reported from 2022 onwards, while including selected earlier studies to provide historical context and illustrate representative structural classes and inhibition mechanisms. Although PTP1B remains an attractive therapeutic target, its clinical validation for diabetes treatment has yet to be achieved. Continued advances in medicinal chemistry and allosteric modulation may help overcome the current translational barriers.

## 1. Introduction

Diabetes mellitus is a chronic metabolic disorder and one of the most pressing global public health challenges. According to the 11th edition (2024) of the International Diabetes Federation (IDF) Diabetes Atlas, approximately 588.7 million adults (20–79 years) are currently living with diabetes, corresponding to 11.1% of the global adult population. Nearly half of affected individuals remain undiagnosed, and projections indicate that the number of cases will rise to 852.5 million by 2050, representing a substantial increase in global prevalence. In addition, a large proportion of the population is at high risk, with hundreds of millions of individuals exhibiting impaired glucose tolerance or impaired fasting glucose. Diabetes is also associated with significant mortality and economic burden, accounting for millions of deaths annually and a considerable share of the global healthcare expenditure [1].

Type 2 diabetes mellitus (T2DM) accounts for over 90% of all cases and is strongly associated with modifiable risk factors, including obesity, physical inactivity, and unhealthy dietary patterns. The rising prevalence of these conditions has contributed to an increasing incidence of T2DM across all age groups, including younger populations. At the pathophysiological level, T2DM is characterized by insulin resistance and progressive β-cell dysfunction, leading to chronic hyperglycemia [2]. Given its multifactorial nature, multiple molecular targets have been explored for therapeutic intervention, including dipeptidyl peptidase-4 (DPP-4) [3], free fatty acid receptor 1 (FFAR1) [4], α-glucosidase [5], peroxisome proliferator-activated receptors (PPARs) [6], sodium–glucose cotransporter 2 (SGLT2) [7], glucagon-like peptide-1 (GLP-1) receptors [8,9] and protein tyrosine phosphatase 1B (PTP1B) [10].

PTP1B, a non-receptor protein tyrosine phosphatase localized to the endoplasmic reticulum (ER), is a key negative regulator of insulin signaling [11]. By dephosphorylating the insulin receptor and its downstream substrates, PTP1B attenuates insulin signaling and contributes to insulin resistance [12]. Experimental evidence supports its central role in metabolic regulation: overexpression of PTP1B induces insulin resistance, whereas its genetic deletion enhances insulin sensitivity, improves glucose tolerance, and confers resistance to diet-induced obesity [13,14].

**Figure 1:**
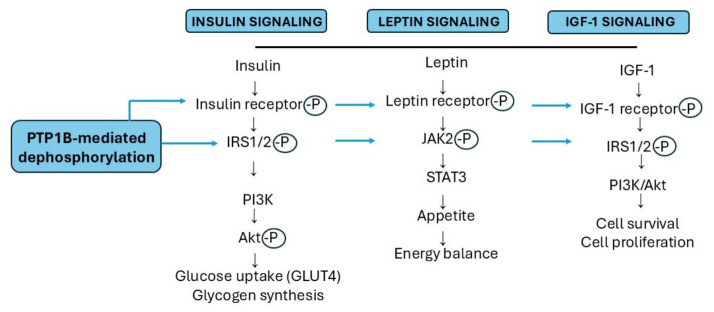
Schematic representation of the principal insulin-, leptin-, and IGF-1-mediated signaling pathways regulated by PTP1B. Blue arrows indicate the major phosphotyrosine-containing signaling proteins that are directly dephosphorylated by PTP1B, including the insulin receptor, IRS1/2, JAK2 and IGF-1 receptor. Through dephosphorylation of these targets, PTP1B negatively regulates glucose homeostasis, energy balance, and cell growth.

Beyond insulin signaling, PTP1B negatively regulates several additional receptor-mediated pathways. One of the best-characterized is leptin signaling, where PTP1B dephosphorylates Janus Kinase 2 (JAK2) associated with the leptin receptor, thereby attenuating hypothalamic leptin signaling and contributing to leptin resistance, obesity, and metabolic dysfunction. Because insulin and leptin signaling converge on several downstream mediators, including insulin receptor substrate (IRS proteins), phosphoinositide 3-kinase (PI3K), and protein kinase B (Akt), PTP1B represents a central molecular node linking glucose homeostasis with energy balance (Figure 1). Notably, it modulates leptin signaling, thereby linking it to obesity and the broader concept of “diabesity” [15]. PTP1B inhibition has also shown therapeutic potential in diabetic complications, including impaired wound healing, where it promotes tissue regeneration and reduces inflammation [16]. Furthermore, increasing evidence suggests a role for PTP1B in neurodegenerative disorders, including Alzheimer’s disease, in which impaired insulin signaling in the brain has led to the concept of “type 3 diabetes” [17]. Dysregulation of PTP1B has also been reported in several cancers, although its role appears to be context-dependent [18]. PTP1B also regulates insulin-like growth factor-1 receptor (IGF-1R) signaling by dephosphorylating activated IGF-1R and hybrid insulin/IGF-1R, thereby modulating cellular growth, proliferation, and survival (Figure 1).

Given its involvement in multiple disease pathways, PTP1B has emerged as a promising therapeutic target. In this review, we provide a comprehensive overview of PTP1B inhibitors, identified by the original numbers cited in the corresponding articles, from both synthetic and natural sources. Synthetic inhibitors reported between 2019 and 2025 are discussed, including compounds targeting the catalytic site, allosteric modulators, and agents acting at the mRNA level. Natural inhibitors identified from 2022 onwards are also presented, complementing the previous literature. Although the primary focus is on natural PTP1B inhibitors reported from 2022 onwards, selected earlier studies have also been included to provide historical context and illustrate representative structural classes and inhibition mechanisms. This review aims to summarize current advances in the field and to highlight the therapeutic potential of PTP1B inhibition in diabetes and related disorders. Unless otherwise stated, the inhibitory activities discussed throughout this review refer to in vitro enzymatic assays.

## 2. PTP1B: Structure, Isoforms, Catalytic Mechanism and Regulation

PTP1B, also known as protein tyrosine phosphatase non-receptor type 1 (PTPN1), is a non-receptor phosphatase that negatively regulates insulin signaling by catalyzing the dephosphorylation of phosphotyrosine (pTyr) residues on the insulin receptor and its downstream substrates. Since its isolation from the human placenta in 1988 by Tonks and co-workers [19], PTP1B has been extensively characterized at the structural level, with the first crystal structure reported in 1994 [20], providing the basis for structure-guided inhibitor design.

PTP1B (1–321 amino acids) is a truncated construct frequently used in structural and biochemical studies because it lacks the C-terminal membrane-targeting domain while retaining full catalytic activity; instead, the physiological enzyme consists of 435 amino acids. Both proteins share the highly conserved N-terminal catalytic domain (residues 30–278), while the full-length enzyme contains a C-terminal extension responsible for anchoring PTP1B to the cytosolic face of the endoplasmic reticulum (ER [21]). This region includes a hydrophobic targeting sequence within the last ~35 residues, which determines subcellular localization and may influence inhibitor accessibility.

**Figure 2:**
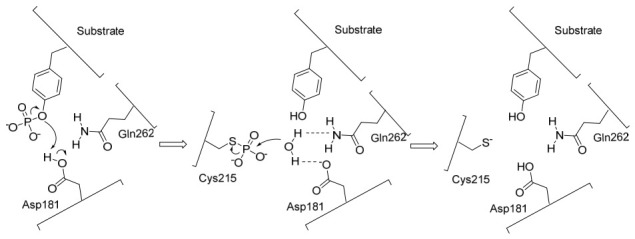
Catalytic pocket of PTP1B and dephosphorylation mechanism.

The catalytic mechanism of PTP1B relies on a highly conserved active site centered on the nucleophilic residue Cys215. Dephosphorylation proceeds through a two-step mechanism involving the formation of a transient phospho-cysteine intermediate. Initially, the thiolate of Cys215 attacks the phosphorus atom of the pTyr substrate, thereby cleaving the pTyr bond. This is followed by hydrolysis of the intermediate, regenerating the active enzyme (Figure 2). The structural organization of the catalytic pocket is well defined and has been extensively exploited for inhibitor design.

Despite being an attractive target, the catalytic site presents significant challenges for drug development. Its high degree of conservation across the protein tyrosine phosphatase (PTP) family limits selectivity, while its strong polarity, required for binding negatively charged phosphate groups, often results in poor membrane permeability of active-site-directed inhibitors. To overcome these limitations, increasing attention has been directed toward adjacent and auxiliary binding regions within the catalytic domain.

Structurally, the catalytic domain can be subdivided into four binding regions: the primary catalytic pocket (site A) and three adjacent subsites (B, C, and D) [22]. Site A is highly conserved and primarily responsible for substrate recognition, making it a challenging target for selective inhibition. In contrast, site B is a broader and less deeply buried pocket that contains both polar and hydrophobic residues including Tyr20, Arg24, Ala27, Phe52, Arg254, Met258 and Gly259, thereby allowing the design of bidentate inhibitors with improved affinity and selectivity. Site C is a relatively flat and solvent-exposed region defined by residues Tyr46, Arg47 and Asp48 which can accommodate negatively charged substituents. Site D is a smaller, partially enclosed pocket comprising Tyr46, Glu115, Lys120, Asp181 and Ser216, and has been shown to contribute significantly to inhibitor potency and selectivity. The coordinated targeting of these regions provides a rational framework for the development of multi-site inhibitors with enhanced drug-like properties [23].

PTP1B activity is further regulated by multiple post-translational modifications, including oxidation, phosphorylation [24,25,26,27] and sumoylation [28], which modulate its catalytic activity and subcellular localization. Reversible oxidation of the active-site cysteine (Cys215) transiently inactivates the enzyme, representing a physiologically relevant mechanism for the regulation of insulin signaling [29,30]. These regulatory processes highlight the conformational flexibility of PTP1B and offer additional opportunities for indirect or allosteric modulation.

## 3. PTP1B Inhibition Strategies and Comparison with LMW-PTP

PTP1B inhibitors that exclusively target the catalytic site (site A) often suffer from limited selectivity and poor cellular permeability. These limitations arise from the need for highly acidic pTyr-mimetic groups to effectively engage the active site, which is characterized by a strongly polar and positively charged environment optimized for binding negatively charged phosphate groups. As a result, active-site-directed inhibitors frequently display suboptimal drug-like properties.

To overcome these challenges, considerable efforts have focused on the design of ligands capable of simultaneously engaging the catalytic site and adjacent non-catalytic regions. This multi-site binding strategy enhances both affinity and selectivity while reducing dependence on highly charged functional groups. Based on their binding modes, PTP1B inhibitors have been classified into several categories, including AC-type (sites A and C), AB-type (sites A and B), ABC-type (sites A, B, and C), and ADC-type (sites A, D, and C) [31].

In parallel, allosteric modulation has emerged as a particularly attractive strategy. Allosteric pockets are generally less conserved and less polar than the catalytic site, offering improved opportunities for achieving selectivity and favorable pharmacokinetic properties. One well-characterized allosteric pocket, located approximately 20 Å from the catalytic site, regulates the interaction between the α7 helix and the α3–α6 helices, which is required for full catalytic activity. Disruption of this interaction results in enzyme inhibition by stabilizing the open, inactive conformation of the WPD (Trp179, Pro180, Asp181) loop, thereby preventing catalysis [32]. A second allosteric region has been identified within a lipophilic pocket formed between β-sheets involving Leu71 and Lys73 and a loop comprising Leu204 and Pro210, structurally connected to the catalytic domain through a short β-strand, enabling long-range conformational communication with the active site. Ligand binding at this site may induce conformational changes that propagate to the active site, thereby modulating catalytic function [33]. Taken together, these observations support a progressive shift in medicinal chemistry strategies from purely active-site inhibition toward multi-site and allosteric approaches, which offer improved prospects for selectivity and drug-like properties.

By exploiting less conserved peripheral regions, these approaches improve selectivity over closely related phosphatases such as T-cell protein tyrosine phosphatase (TCPTP). TCPTP deserves particular attention because it has overlapping biological functions, shares approximately 72% sequence identity (86% similarity) with PTP1B in the catalytic domain [34], making selective active-site inhibition challenging and potentially associated with off-target effects. Instead, although high selectivity has traditionally been regarded as a desirable feature of PTP1B inhibitors, dual inhibition of PTP1B and TCPTP has recently been proposed as an alternative strategy to overcome compensatory signaling and enhance metabolic efficacy and may partially compensate for PTP1B inhibition under certain physiological or pathological conditions. Nevertheless, this approach requires careful evaluation because TCPTP plays essential roles in hematopoiesis and immune regulation, raising concerns regarding potential adverse effects associated with reduced selectivity.

PTP1B belongs to the PTP superfamily, a diverse group of enzymes that catalyze the dephosphorylation of pTyr residues [35]. Within this family, PTP1B is classified as a Class I classical non-receptor PTP. In contrast, low molecular weight PTP (LMW-PTP) is a ~18 kDa enzyme belonging to Class II PTPs [36]. LMW-PTP is encoded by the acid phosphatase 1 (ACP1) gene and exists as two major isoforms, IF1 and IF2, which arise from alternative mRNA processing and differ in their kinetic and regulatory properties [37]. Among the two isoforms, IF1 has been strongly associated with insulin resistance and glucose homeostasis, making it particularly relevant in the context of diabetes therapy. Although classified into distinct PTP classes, both enzymes share a cysteine-dependent catalytic mechanism and are regulated by reversible redox processes involving the active-site cysteine residue. The catalytic core of LMW-PTP is defined by a triad comprising Cys12, Arg18, and Asp129, which are essential for pTyr hydrolysis [38]. Enzymatic activity is modulated by reversible oxidation of Cys12, leading to transient inactivation through disulfide bond formation with Cys17 [36]. This redox-dependent regulation is analogous to that observed for PTP1B and represents a conserved mechanism among cysteine-based phosphatases.

From a structural standpoint, LMW-PTP differs from PTP1B by its smaller size and more compact architecture, resulting in fewer well-defined auxiliary binding pockets [39]. While PTP1B possesses multiple adjacent binding regions that enable multi-site and allosteric targeting, LMW-PTP presents a more constrained surface, which may influence inhibitor design strategies. Nevertheless, LMW-PTP plays a significant role in both metabolic regulation and cancer progression. It has been implicated in insulin receptor signaling, with knockout or downregulation studies demonstrating improved insulin sensitivity and glycemic control [40]. In addition, increased expression and activity of LMW-PTP have been reported in several malignancies and are associated with tumor progression and poor prognosis [41,42,43].

Overall, while PTP1B remains the most extensively investigated target within the PTP family, LMW-PTP represents a complementary and still underexplored therapeutic opportunity. A deeper understanding of the structural, mechanistic and functional differences between these phosphatases may support the rational development of next-generation inhibitors with improved selectivity and therapeutic potential. The inclusion of LMW-PTP in this review reflects its emerging role as a complementary therapeutic target sharing mechanistic similarities with PTP1B and providing additional opportunities for phosphatase-directed drug discovery.

These observations further highlight the delicate balance between achieving high target selectivity and preserving therapeutic efficacy, which remains one of the major challenges in the development of next-generation PTP1B inhibitors.

## 4. LMW-PTP Inhibitors: SPAA-Derived Compounds and Structure–Activity Relationships

To date, only a limited number of LMW-PTP inhibitors have been reported, often characterized by insufficient potency and selectivity, which has hindered their development as therapeutic agents.

**Figure 3:**
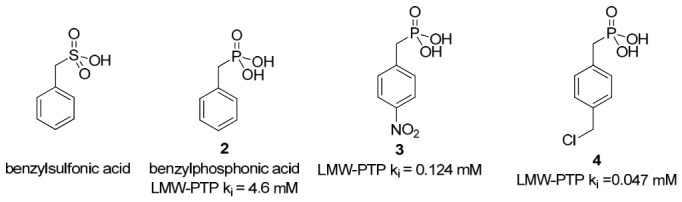
Representative structures of sulfonic and phosphonic derivatives as LMW-PTP inhibitors.

Fonseca and co-workers described sulfonic and phosphonic acid derivatives as LMW-PTP inhibitors, with phosphonic acids emerging as the more favorable class, acting as competitive inhibitors [39]. Notably, sulfonic and phosphonic acid derivatives differ substantially in acidity, with sulfonic acids being significantly more acidic than phosphonic acids. This difference affects ionization at physiological pH and consequently binding affinity, selectivity, and membrane permeability. Compound **2** represents the first reported inhibitor of human LMW-PTP and served as a lead scaffold for the development of derivatives **3** and **4**, which displayed improved inhibitory potency (Figure 3).

**Figure 4:**
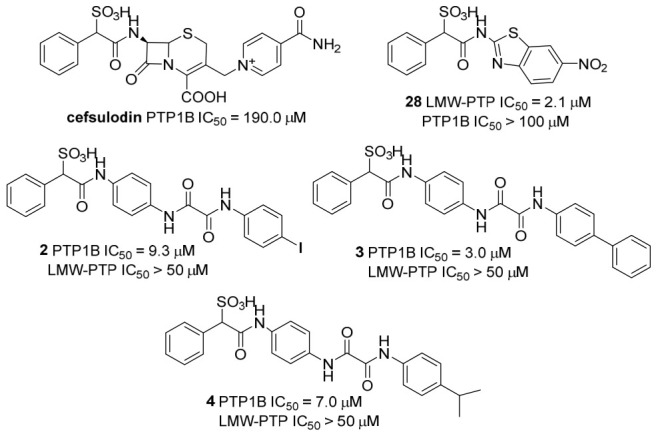
Representative structures with the sulfonylphenylacetamide (SPAA) scaffold obtained from cefsulodin.

A significant advancement in this area emerged from studies on cefsulodin (Figure 4), third generation β-lactam antibiotic, which was found to be a PTP1B inhibitor. The sulfonylphenylacetamide (SPAA) fragment was subsequently identified as a novel pTyr mimetic and a key pharmacophoric element for LMW-PTP selectivity [44]. The SPAA scaffold provided a less classical alternative to phosphate-based mimetics, offering improved opportunities for structural diversification while retaining the ability to interact with the positively charged catalytic pocket.

Subsequent high-throughput screening of SPAA-based libraries led to the identification of a series of derivatives exhibiting enhanced potency and a measurable degree of selectivity over other PTP family members [45]. Among these, compound **28** (Figure 4) demonstrated at least 50-fold selectivity for LMW-PTP over 24 other PTPs, highlighting the potential of fragment-based and scaffold-optimization strategies in overcoming selectivity barriers within this enzyme class.

Importantly, additional derivatives (compounds **2**–**4**, Figure 4), generated through systematic functionalization of the SPAA core with structurally diverse linkers to increase chemical diversity, displayed significant inhibitory activity toward PTP1B rather than LMW-PTP [44]. This observation underscores the delicate balance between subtle structural modifications and isoform selectivity within closely related phosphatases. Minor variations in linker length, orientation, or substituent distribution may redirect binding preference from one phosphatase subtype to another, emphasizing the importance of precise structure–activity relationship (SAR) studies.

Overall, the SPAA-based approach illustrates how fragment-based design and targeted chemical diversification can provide a viable pathway toward selective inhibition of LMW-PTP, although further optimization is required to achieve drug-like properties compatible with therapeutic development.

**Figure 5:**
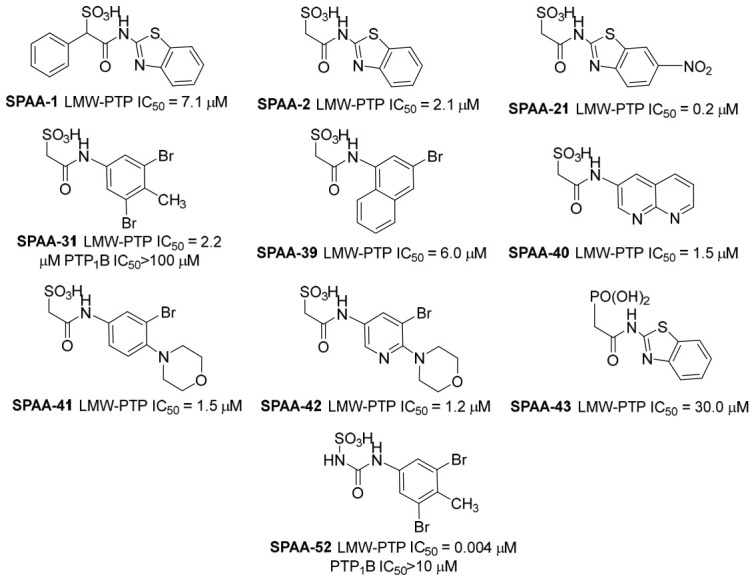
Representative structures of SPAA derivatives synthesized by He and coworkers.

Starting from **SPAA-1**, He and co-workers performed systematic structural modifications on the different molecular regions, including the sulfonic acid moiety, the aromatic phenyl ring, the linker, and the benzothiazole fragment, which were variously substituted or replaced (Figure 5). This comprehensive SAR study provided key insights into the structural determinants governing potency and selectivity toward LMW-PTP [46]. Remarkably, the phenyl ring was found to be non-essential for activity, as **SPAA-2** exhibited slightly improved potency compared to **SPAA-1**. Crystallographic comparison of the **SPAA-1**–LMW-PTP and **SPAA-2**–LMW-PTP complexes revealed that both ligands bind to the same active-site region and interact with similar residues, indicating that removal or modification of the phenyl moiety does not significantly alter the binding mode [46]. This finding highlights the robustness of the core pharmacophoric interactions within the catalytic pocket. Substitutions on the benzothiazole ring were partially tolerated, while electron-withdrawing substituents were acceptable, with the nitro derivative (**SPAA-21**, Figure 5) showing a modest increase in potency. More extensive scaffold modifications, including replacement of the benzothiazole ring with 3,5-dibromo-4-methyl (**SPAA-31**), bromonaphthyl (**SPAA-39**), 1,8-naphthyridine (**SPAA-40**), bromophenyl-morpholine (**SPAA-41**), or 4-(3-bromopyridin-2-yl)morpholine (**SPAA-42**), were generally tolerated but did not result in significant improvements in inhibitory potency (Figure 5). These results suggest that while the distal aromatic system contributes to binding stabilization, it is not the primary driver of affinity.

In contrast, the sulfonic acid group proved to be a critical pharmacophoric element. Bioisosteric replacement of the sulfonic acid was largely not tolerated, underscoring its essential role in mimicking the phosphate group and engaging the positively charged catalytic pocket. The only acceptable substitution was phosphonic acid (**SPAA-43**, Figure 5), which retained partial inhibitory activity. Similarly, elongation of the linker by insertion of two or three methylene groups was detrimental, indicating a strict spatial requirement for optimal positioning of the pharmacophore within the active site [46]. A breakthrough was achieved with the modification of **SPAA-31**, where the replacement of the methylene linker with an NH group generated **SPAA-52** (Figure 5). This derivative displayed a dramatic increase in potency (IC_50_ = 0.004 µM), attributed to the introduction of a urea functionality capable of forming a bidentate hydrogen bond with Asp129. This additional interaction significantly stabilizes the enzyme–inhibitor complex and accounts for the observed potency enhancement. Notably, **SPAA-52** (Figure 5) represents the most selective compound within this series, exhibiting more than 8000-fold selectivity over 24 other phosphatases and acting as a highly potent LMW-PTP inhibitor (K_i_ = 1.2 nM) [46]. These findings elegantly demonstrate how precise manipulation of hydrogen-bonding capabilities and spatial orientation within the catalytic pocket can dramatically enhance both potency and selectivity, even within highly conserved phosphatase families.

**Figure 6:**
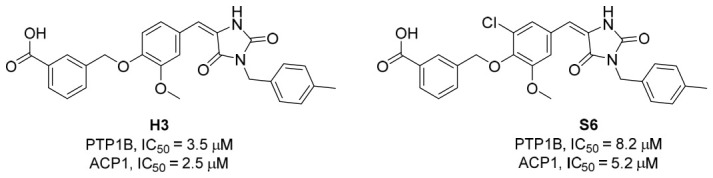
Representative structures of PTP1B and ACP1 dual inhibitors.

In addition to selective inhibitors, the development of dual inhibitors has also been explored. Virtual screening of the ChEMBL database led to the identification of dual inhibitors that, although exhibiting relatively low potency, showed stronger inhibitory effects toward PTP1B than toward LMW-PTP IF1, the isoform most closely associated with insulin resistance and metabolic regulation. Among the analyzed derivatives, compounds **H3** and **S6**, both characterized by a hydantoin (imidazolidine-2,4-dione) scaffold, exhibited moderate dual inhibitory activity. The presence of this heterocyclic core may contribute to phosphatase recognition through its hydrogen-bonding capabilities and favorable geometric arrangement. Derivative **H3** showed IC_50_ values of 3.5 µM for PTP1B and 2.5 µM for ACP1, whereas **S6** displayed IC_50_ values of 8.2 µM for PTP1B and 5.2 µM for ACP1 (Figure 6) [47].

## 5. PTP1B Inhibitors from Natural Sources

Natural products have been extensively reviewed as a rich source of PTP1B inhibitors, and the reader is referred to recent comprehensive studies for detailed classifications and biological profiles [48,49,50,51]. Woo and co-workers classified approximately 500 natural PTP1B inhibitors into five major chemical classes: fatty acids, phenolic derivatives, terpenoids, steroids, and alkaloids, isolated from nearly 100 different species [52].

Natural products represent a structurally diverse source of PTP1B modulators, providing unique scaffolds capable of interacting with both catalytic and allosteric sites. Their chemical scaffolds, ranging from terpenoids and flavonoids to lignans, catechins, and coumarins, provide valuable templates for identifying novel inhibitory frameworks. Compared to classical pTyr mimetics, many natural compounds display moderate lipophilicity and structural complexity, enabling interaction with peripheral binding regions and potentially improving selectivity and membrane permeability. Despite these advantages, natural inhibitors generally exhibit moderate potency and heterogeneous inhibition mechanisms, including competitive, non-competitive, and mixed modes.

From a drug discovery perspective, the principal value of natural products lies in their role as lead scaffolds. Structure-guided optimization and semi-synthetic modification can transform these bioactive frameworks into more potent and selective derivatives. Natural compounds acting through allosteric or multi-site mechanisms provide important validation of alternative binding regions beyond the highly conserved catalytic pocket.

**Figure 7:**
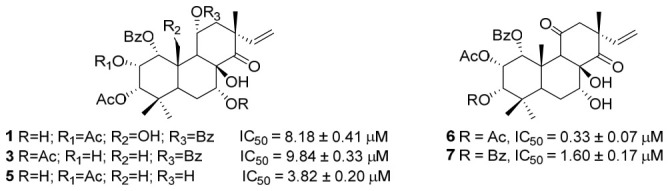
Representative structures of pimarane-type diterpenes isolated from *Orthosiphon stamineus*.

Among reported examples, pimarane-type diterpenes isolated from *Orthosiphon stamineus* exhibited inhibitory activity against PTP1B, with IC_5__0_ values ranging from submicromolar to low micromolar levels and diverse inhibition mechanisms (Figure 7) [53]. Compound **1** (IC_50_ = 8.18 ± 0.41 µM) showed mixed competitive inhibition, whereas compounds **3** and **5** (IC_50_ = 9.84 ± 0.33 µM and 3.82 ± 0.20 µM, respectively) displayed non-competitive inhibition, suggesting a possible allosteric binding mode. Compound **6**, the most potent derivative (IC_50_ = 0.33 ± 0.07 µM), acted as a competitive inhibitor, while compound **7** (IC_50_ = 1.60 ± 0.17 µM) exhibited uncompetitive inhibition [53].

**Figure 8:**
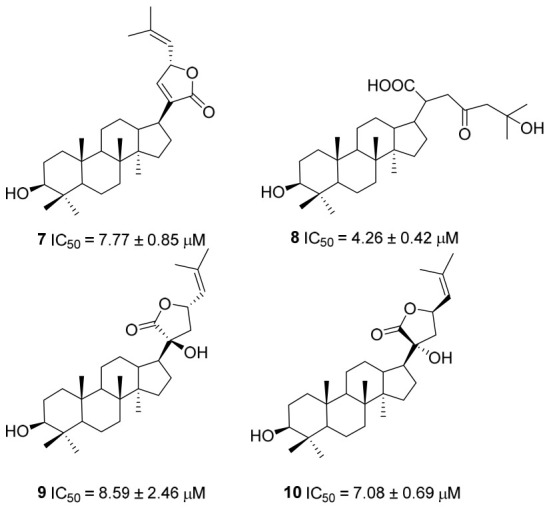
Representative structures of dammarane-type triterpenes isolated from *Gymnostemma pentaphyllum*.

Among the terpenoid class, dammarane-type triterpenoids isolated from *Gynostemma pentaphyllum* have also been reported as PTP1B inhibitors. In particular, compounds **7**–**10** (Figure 8) exhibited inhibitory activity with IC_50_ values ranging from 4.26 to 8.59 µM and displayed different inhibition mechanisms. Compounds **7** and **8** acted as competitive inhibitors, whereas derivatives **9** and **10** showed mixed-type inhibition, suggesting that subtle structural variations within the dammarane scaffold may influence both potency and binding mode [54].

**Figure 9:**
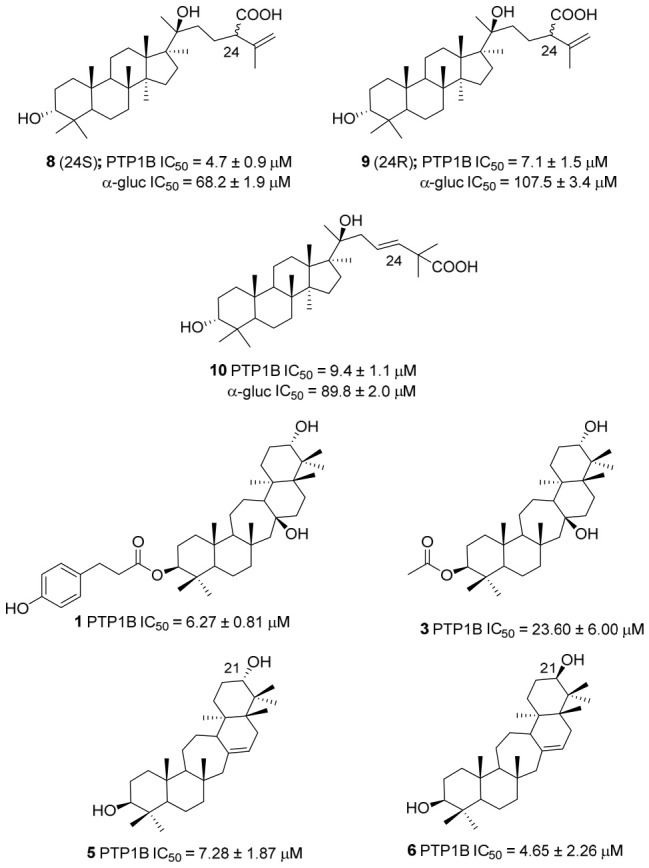
Representative structures of dammarane-type triterpenes isolated from *Aglaia perviridis* and serratane-type triterpenes isolated from *Huperzia serrata*.

Structurally related dammarane triterpenoids have also been isolated from *Aglaia perviridis*. Notably, compounds **8**–**10** (Figure 9) exhibited dual inhibitory activity against both PTP1B (IC_50_ = 4.7–9.4 µM) and α-glucosidase (IC_50_ = 68.2–107.5 µM), highlighting the potential of this scaffold for multitarget antidiabetic drug design [55].

Another noteworthy class comprises serratane-type triterpenoids, pentacyclic triterpenes characterized by a seven-membered C ring, isolated from *Huperzia serrata*. Compounds **1**, **3**, **5**, and **6** (Figure 9) displayed significant PTP1B inhibitory activity, with IC_50_ values ranging from 4.65 to 23.60 µM, and all acted as competitive inhibitors [56]. Structure–activity relationship analysis indicated that compound **1** was more potent than compound **3**, suggesting that the presence of a 3*β*-dihydro-*p*-coumaroyl substituent enhances inhibitory activity. Furthermore, comparison of compounds **5** (21*β*-OH, IC_50_ = 7.28 ± 1.87 μM) and **6** (21*α*-OH, IC_50_ = 4.65 ± 2.26 µM) demonstrated that the stereochemical configuration of the hydroxyl group at C-21 significantly influences PTP1B inhibition [56].

**Figure 10:**
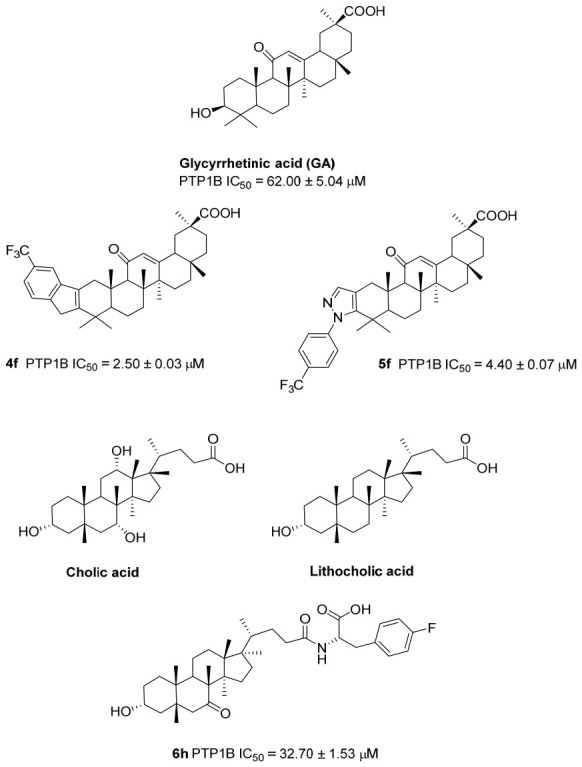
Representative structures of pentacyclic triterpenes isolated from *Glycyrrhiza glabra* and cholic acid derivatives.

Glycyrrhetinic acid (**GA**), a pentacyclic triterpene isolated from *Glycyrrhiza glabra*, which is itself a weak PTP1B inhibitor (IC_50_ = 62.00 ± 5.04 µM), has served as a valuable lead scaffold for the development of more potent derivatives. Two series of analogs were synthesized by fusing either an indole or an *N*-phenylpyrazole ring to ring A of the glycyrrhetinic acid scaffold. Among the synthesized compounds, derivative **4f** (Figure 10) exhibited the highest inhibitory potency (IC_50_ = 2.50 ± 0.03 µM), followed by derivative **5f** (IC_50_ = 4.40 ± 0.07 µM). Both compounds acted as non-competitive inhibitors of PTP1B, suggesting interaction with an allosteric binding site rather than the catalytic pocket. Compared with the parent compound, the fused-ring derivatives displayed approximately 25-fold and 14-fold improvements in potency, respectively, demonstrating that rigidification and expansion of the aromatic framework around ring A markedly enhance PTP1B inhibition [57].

In addition to triterpenoids, several naturally occurring steroids have been reported to exhibit moderate inhibitory activity against PTP1B. These include bile acids such as cholic acid and lithocholic acid, as well as trodusquemine, a naturally occurring aminosterol that represents the first PTP1B inhibitor to advance into clinical trials for the treatment of diabetes. Owing to its unique allosteric mechanism of action and clinical relevance, trodusquemine is discussed in detail in Section 8.

Inspired by the moderate PTP1B inhibitory activity of cholic and lithocholic acids, a series of semisynthetic steroid derivatives was subsequently developed. Among these, compound **6h** (Figure 10) exhibited the most favorable inhibitory profile, showing good potency together with approximately seven-fold selectivity over TCPTP. Kinetic studies demonstrated that compound **6h** acts as a reversible mixed-type inhibitor, further supporting steroidal scaffolds as attractive starting points for the development of selective PTP1B inhibitors [58].

**Figure 11:**
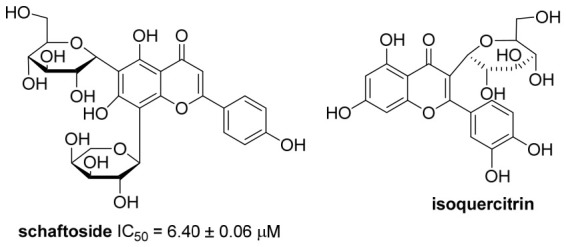
Representative structures of C-glycosyl flavonoids.

Schaftoside (Figure 11), a C-glycosyl flavonoid isolated from *Eleusine indica* and obtained from fermented wheat, exhibits inhibitory activity against PTP1B (IC_50_ = 6.40 ± 0.06 µM) [59]. Its structure combines a polyphenolic core, capable of hydrogen bonding and π-πinteractions, with a sugar moiety that may influence solubility and binding orientation within the enzyme pocket. These features make flavonoid glycosides attractive multifunctional scaffolds for further optimization. Interestingly, isoquercitrin (Figure 11), a structurally related analog isolated from *Bidens bipinnata*, contains a single glycosidic moiety and therefore exhibits lower polarity. Preliminary studies have also shown inhibitory activity of isoquercitrin against PTP1B [60].

**Figure 12:**
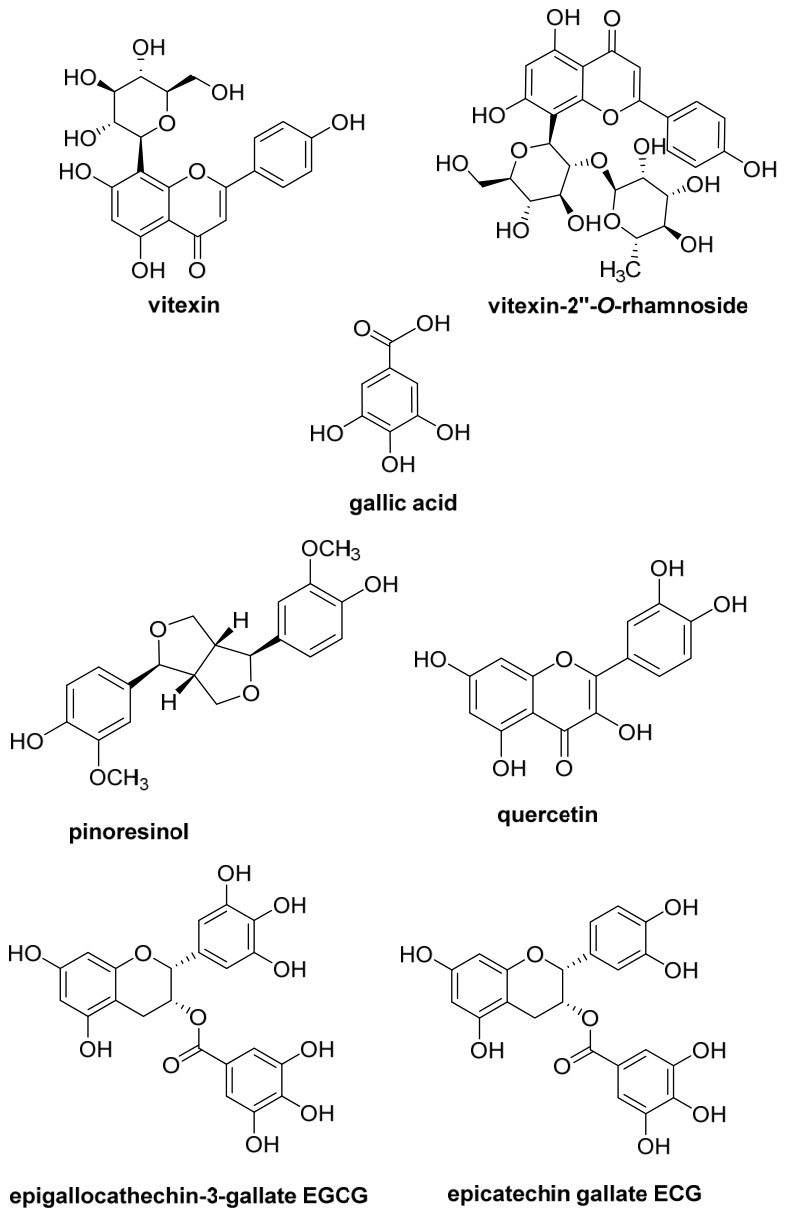
Representative structures of phenolic compounds.

Fermentation processes have also been shown to enhance the phenolic content of cereals, thereby increasing their potential bioactivity. In fermented millet, elevated levels of phenolic compounds have been reported, and partial PTP1B inhibitory activity has been attributed to vitexin and vitexin 2″-*O*-rhamnoside (Figure 12) [61]. The study of fermented millet is supported by both traditional dietary practices in Asian and African populations and epidemiological observations indicating a lower incidence of T2DM in populations with regular millet consumption [62]. These findings suggest a potential link between diet-derived phenolic compounds and the modulation of metabolic targets such as PTP1B.

Similarly, phenolic extracts obtained from whole dehulled Indian red rice have shown PTP1B inhibitory activity [63]. Comparable effects have been reported for black and green currant extracts, where gallic acid (Figure 12), the most abundant phenolic constituent, was identified as the primary contributor to the observed activity [64]. Gallic acid, a small polyphenolic molecule rich in hydroxyl groups, can form multiple hydrogen-bonding interactions within the catalytic pocket, although its high polarity may limit membrane permeability.

Phenolic extracts derived from cold-pressed grape seed oils of different cultivars have also been evaluated for their PTP1B inhibitory potential. Their activity was found to correlate with total phenolic content, particularly with the presence of pinoresinol and quercetin (Figure 12), identified as key contributors [65]. Structurally, lignans such as pinoresinol and flavonols, such as quercetin, provide rigid aromatic frameworks that may interact with peripheral or allosteric regions of PTP1B, potentially offering advantages over highly charged phosphate mimetics. Overall, these observations highlight the relevance of diet-derived phenolic compounds as mild yet structurally diverse PTP1B modulators. Although their intrinsic potency is generally lower than that of optimized synthetic inhibitors, their favorable safety profiles and structural plasticity make them attractive starting points for semi-synthetic modification and structure-guided optimization.

Regular consumption of tea has been associated with a reduced risk of chronic degenerative diseases, including diabetes [66]. Green tea is rich in phenolic compounds and has been reported to exert significant metabolic benefits. Its major bioactive constituents are catechins, among which epigallocatechin-3-gallate (EGCG, Figure 12) is the most abundant, with a single cup containing up to 200 mg. EGCG has been shown to improve glucose homeostasis and suppress hepatic gluconeogenesis and lipogenesis, while also exerting beneficial effects on diabetic ulcers through anti-inflammatory and wound-healing activities [67]. These pleiotropic effects suggest that catechins may modulate multiple pathways relevant to insulin sensitivity and tissue repair.

Both hot and cold tea extracts have been evaluated for inhibitory activity against PTP1B and LMW-PTP, with composition and potency depending on extraction conditions. Among the identified catechins, only EGCG and epicatechin gallate (ECG, Figure 12) exhibited significant inhibitory activity against both enzymes. Mechanistically, EGCG acts as a mixed-type, non-competitive inhibitor capable of binding to multiple sites, with a reported K_i_ value of 26 nM [68]. Its polyphenolic structure, characterized by multiple hydroxyl groups and a gallate moiety, enables extensive hydrogen bonding and π-π interactions with both catalytic and peripheral regions. This multi-site binding capability likely underlies its mixed inhibition profile. Despite its relatively high potency in enzymatic assays, EGCG exhibits suboptimal drug-like properties due to high polarity and metabolic instability. Nevertheless, its multi-target activity and favorable safety profile make it an attractive lead scaffold for semi-synthetic optimization aimed at improving stability, permeability, and selectivity.

**Figure 13:**
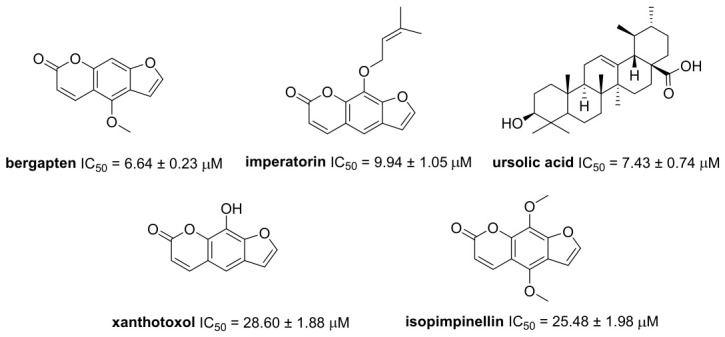
Representative structures of coumarin derivatives isolated from *Ammi majus*.

Several coumarin derivatives have been isolated from *Ammi majus* (Bishop’s weed), an herbaceous plant widely distributed in Europe, Asia, and Africa. Among these, bergapten, imperatorin, xanthotoxol, and isopimpinellin have shown inhibitory activity against PTP1B (Figure 13). Bergapten and imperatorin displayed the highest potency, with IC_50_ values of 6.64 ± 0.23 µM and 9.94 ± 1.05 µM, respectively, comparable to the reference compound ursolic acid (IC_50_ = 7.43 ± 0.74 µM). Kinetic studies indicated that these compounds act as non-competitive inhibitors, binding to an allosteric site rather than to the catalytic pocket with K_i_ values of 6.73 µM (bergapten) and 8.44 µM (imperatorin) [69]. From a drug discovery perspective, this non-competitive mechanism is particularly relevant, as targeting allosteric sites may help overcome selectivity limitations associated with the highly conserved catalytic domain. This is especially important considering the close homolog TCPTP, which shares high sequence identity within the catalytic domain [34], making selective active-site inhibition challenging and potentially associated with off-target effects.

The planar aromatic structure and moderate lipophilicity of coumarins favor interaction with hydrophobic or partially solvent-exposed allosteric pockets. Their scaffold therefore provides opportunities for structural diversification aimed at improving affinity and selectivity while maintaining favorable permeability compared to highly charged phosphate mimetics. Coumarin derivatives thus represent attractive starting points for the development of selective allosteric PTP1B inhibitors.

Natural products frequently exhibit dual or multi-target activity, a feature that may represent both an advantage and a limitation in drug development. Many natural PTP1B inhibitors also modulate additional targets involved in glucose homeostasis and insulin sensitivity, such as α-glucosidase, PPAR-γ, and other metabolic regulators. This pleiotropic activity may enhance antidiabetic efficacy through synergistic mechanisms, but it can also complicate mechanistic interpretation and selectivity profiling, particularly when assessing the specific contribution of PTP1B inhibition to the overall biological effect.

## 6. Synthetic and Multitarget PTP1B Inhibitors

### 6.1. Synthetic PTP1B Inhibitors

Synthetic PTP1B inhibitors commonly combine polycyclic or heteroaromatic scaffolds with acidic or polar functionalities, designed to interact with the positively charged catalytic pocket. Comprehensive overviews of synthetic PTP1B inhibitor classes reported up to 2020 are available in previous reviews [10,70].

#### 6.1.1. Thiazolidine-2,4-dione (TZD) and Imidazolidine-2,4-dione (IZD) Derivatives

**Figure 14:**
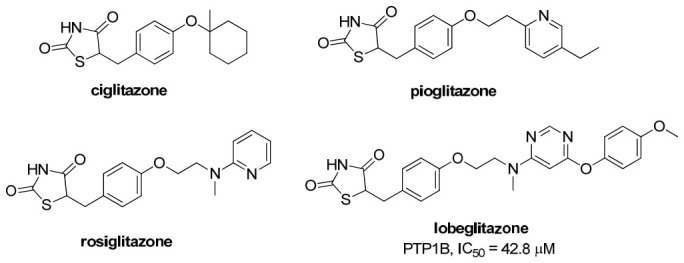
Representative structures of thiazolidine-2,4-dione (TZD) derivatives.

The thiazolidine-2,4-dione (TZD) scaffold is widely used in oral antidiabetic agents for the treatment of T2DM, mainly through activation of PPAR-γ, which improves insulin sensitivity and reduces hepatic glucose production [71]. Classic TZD drugs include ciglitazone, rosiglitazone, pioglitazone and lobeglitazone (Figure 14). Although the clinical use of some TZDs has been limited by safety concerns, their pharmacophoric relevance has stimulated interest in their repurposing and structural modification [72]. Lobeglitazone, a PPAR-γ agonist approved in South Korea for T2DM, was also reported to inhibit PTP1B, with an IC_50_ value of 42.8 µM, acting as a non-competitive inhibitor [73]. Although its potency toward PTP1B is moderate, this dual activity highlights the potential of established antidiabetic scaffolds for multitarget modulation.

**Figure 15:**
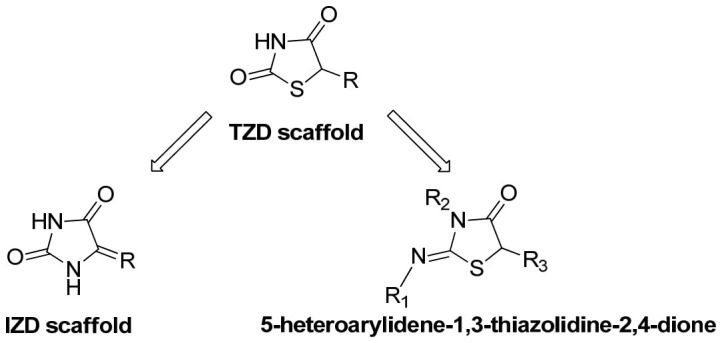
Modification of the thiazolidine-2,4-dione (TZD) scaffold.

The TZD core has been isosterically transformed into the imidazolidine-2,4-dione (IZD, hydantoin) ring system, which displays lower acidity while retaining hydrogen-bonding potential. Additional modification included replacement of the 2-oxo group with an imino functionality, generating analogs with altered electronic distribution and hydrogen-bonding properties (Figure 15) [74]. In these derivatives, the Z configuration around the heteroarylidene double bond was essential for effective bidentate inhibition, likely ensuring the correct spatial orientation of pharmacophoric groups toward the catalytic site and adjacent non-catalytic regions.

**Figure 16:**
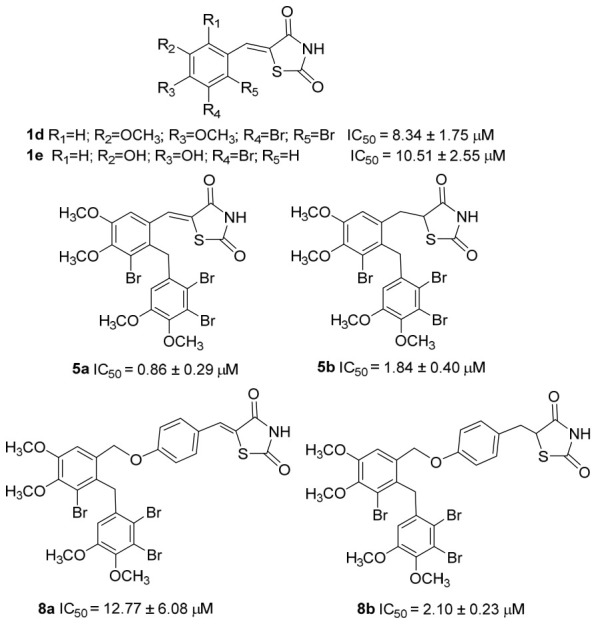
Representative structures of TZD-containing compounds with bromophenolic fragments.

Chemical modification of natural product-inspired scaffolds has also been applied to TZD and IZD derivatives. Wang and co-workers functionalized these cores with mono or dibenzyl bromophenol moieties inspired by bromophenolic compounds isolated from red algae of the *Rhodomelaceae* family, previously known for α-glucosidase inhibition [75,76]. This hybrid design aimed to combine the insulin-sensitizing TZD/IZD scaffold with bromophenolic fragments to generate multifunctional antidiabetic candidates. Among the monobenzyl derivatives, only TZD-containing compounds showed significant PTP1B inhibition, with derivatives **1d** and **1e** displaying IC_50_ values of 8.34 ± 1.75 µM and 10.51 ± 2.55 µM, respectively. In the dibenzyl series, several TZD derivatives showed improved potency, with IC_50_ values ranging from 0.86 to 12.77 µM, particularly compounds **5a**, **5b**, **8a**, and **8b** (Figure 16) [77]. From a SAR perspective, the bromophenolic substituents may enhance hydrophobic and halogen-bonding interactions within peripheral regions, while the dibenzyl arrangement may favor multi-site engagement.

#### 6.1.2. Allosteric Imidazolidinone Derivatives

Ottanà and co-workers synthesized 3-aryl-5-arylidene-2-thioxo-4-imidazolidinone derivatives as allosteric modulators of PTP1B targeting the second allosteric site (Table 1) [78]. This lipophilic pocket, located between *β*-sheets and a loop, is structurally connected to the catalytic domain. The 5-arylidene moiety was designed to occupy the hydrophobic cavity, promoting Van der Waals and π–π interactions, while aromatic substituents at the N3 position enhanced hydrophobic anchoring. The imidazolidinone NH group was retained to allow hydrogen-bonding interactions at the pocket interface. Kinetic studies confirmed that these compounds act as non-competitive inhibitors, consistent with allosteric binding [78].

Selected derivatives showed low-micromolar activity, including compounds **7a** and **7c** (IC_50_ = 3.5 µM), **7e** (IC_50_ = 2.9 µM), and **7f** (IC_50_ = 2.7 µM) (Table 1). These compounds illustrate how selective engagement of distal lipophilic pockets can modulate PTP1B activity without direct catalytic-site competition.

#### 6.1.3. Chromone and TZD-Based Hybrids

The benzo-γ-pyrone core of flavonoids has been widely explored as a scaffold for PTP1B inhibition. This pharmacophoric motif is also found in several naturally occurring PTP1B inhibitors discussed above, including the flavonoid glycosides schaftoside and isoquercitrin (Figure 11), as well as phenolic derivatives such as quercetin and related compounds (Figure 12). The recurrent occurrence of the benzo-γ-pyrone scaffold in natural PTP1B inhibitors has inspired the development of numerous synthetic analogs aimed at improving potency, selectivity, and drug-like properties. Among these approaches, the chromone moiety was hybridized with a TZD group through an amide linker, a structural element previously incorporated into PTP1B inhibitors [79,80]. The amide linker provides conformational flexibility, hydrogen-bonding capability, and appropriate spatial orientation between pharmacophoric fragments. Consequently, chromone–TZD hybrids represent a rational scaffold combination, integrating a naturally inspired aromatic core with a known antidiabetic pharmacophore.

Chromone derivatives synthesized by Zheng and co-workers showed significant PTP1B inhibitory activity, with IC_5__0_ values ranging from 1.40 to 16.83 µM (Table 2) [81]. Compound **9** was the most potent derivative, with an IC_50_ value of 1.40 ± 0.04 µM and approximately 19-fold selectivity over TCPTP. SAR analysis indicated that both the electronic and positional effects influenced activity: *para*-methyl (**9**) and *para*-methoxy (**10**) substituents enhanced potency, while among electron-withdrawing groups (EWG), the *meta*-nitro derivative (**21**) was particularly favorable. These findings highlight the importance of fine-tuning electronic properties and substitution patterns to achieve improved potency and selectivity.

#### 6.1.4. TZD–Thiadiazole Hybrids

The TZD moiety has also been employed as a head group to engage the catalytic site of PTP1B, and conjugated to a 1,3,4-thiadiazole linker and a substituted aromatic tail. The 1,3,4-thiadiazole ring is a privileged scaffold in medicinal chemistry and appears in several bioactive compounds [82].

**Figure 17:**
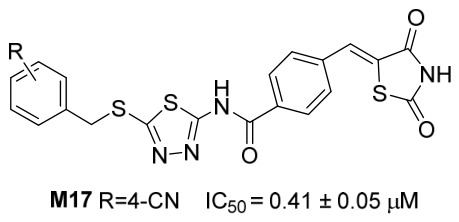
Representative structures of TZD–Thiadiazole Hybrids.

The resulting hybrid compounds displayed significant PTP1B inhibition, with IC_50_ values ranging from 0.41 ± 0.05 to 4.68 ± 0.44 µM [83]. Among them, compound **M17** was identified as a non-competitive inhibitor of PTP1B (Figure 17). Structurally, the TZD head group may contribute to catalytic-pocket anchoring, while the thiadiazole linker and aromatic tail may extend toward secondary regions, enabling conformational modulation or peripheral site-engagement. Notably, the inhibitory activity is strongly influenced by the position of substituents on the aromatic ring, with *para*-substituted derivatives generally exhibiting the highest potency, followed by *ortho*- and *meta*-substituted analogs.

#### 6.1.5. Computer-Aided Design and Virtual Screening

**Figure 18:**
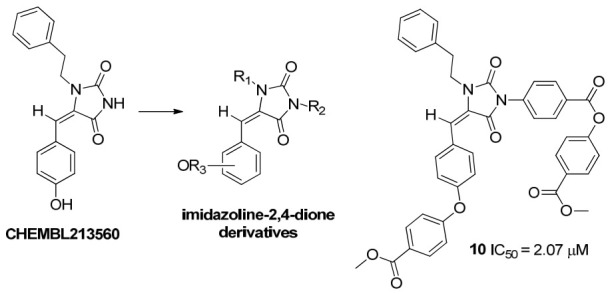
Representative structures of CADD-derived PTP1B inhibitors identified by Wang and co-workers through virtual screening.

Computer-aided drug design (CADD) has been widely applied to the discovery of PTP1B inhibitors. Using a CDOCKER-based virtual screening protocol, Wang and co-workers screened ZINC, NCI, and PubChem databases and identified **CHEMBL213560** as a lead compound. Structural optimization generates a virtual library of approximately 22,500 IZD derivatives, from which 15 compounds were synthesized and evaluated. Compound **10** showed the highest activity with an IC_50_ value of 2.07 µM (Figure 18) [84]. Notably, the IZD core is also found in the dual PTP1B/ACP1 inhibitors **H3** and **S6** (Figure 6), highlighting its potential as a versatile scaffold for phosphatase inhibitor design.

**Figure 19:**
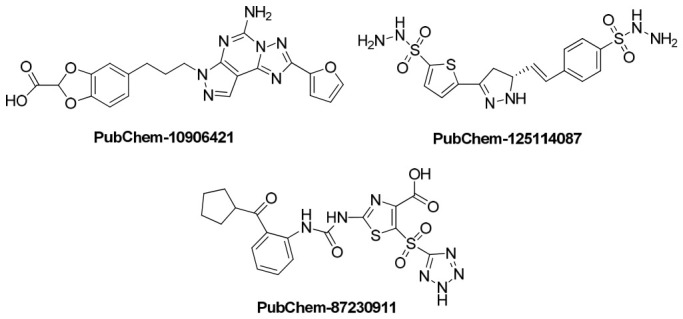
Representative structures of PTP1B inhibitor candidates identified by Zhao and co-workers through virtual screening.

Zhao and co-workers performed virtual screening across PubChem, ChEMBL, and ZINC, covering approximately 108 million compounds, aiming to identify inhibitors with reduced anionic character, improved oral bioavailability, and the ability to engage both catalytic and proximal non-catalytic sites [85]. Three candidates were proposed: **PubChem-10906421**, **PubChem-12514087**, and **PubChem-87230911** (Figure 19). However, no enzymatic validation was reported, which limits the translational significance of these findings and emphasizes the need to combine in silico approaches with biochemical and cellular assays.

#### 6.1.6. Furanocoumarin Derivatives

**Figure 20:**
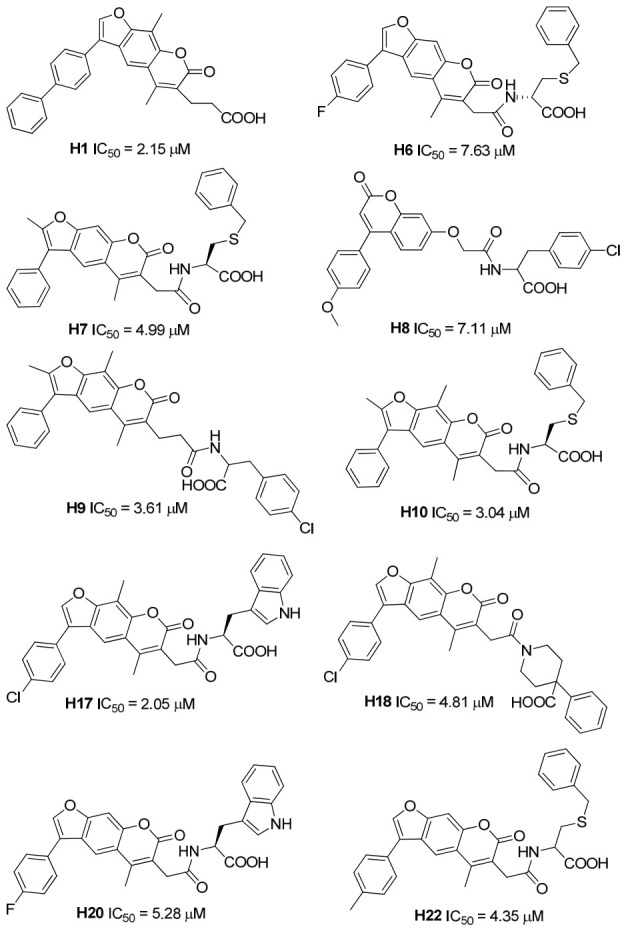
Representative structures of furanocoumarin derivatives.

Using natural product libraries and combined ligand- and structure-based virtual screening, Yang and co-workers identified 26 structurally diverse derivatives as potential PTP1B inhibitors [86]. The most active compounds shared a carboxylic acid group and a coumarin scaffold: notably, nine of the ten most active compounds contained a furanocoumarin core (Figure 20). SAR analysis indicated that variability in the side chains of the 2*H*-pyran-2-one ring was tolerated, while methyl substitution on either the furan or the benzene ring enhanced activity. Docking suggested that the carboxylic acid group occupies the catalytic site through hydrogen-bonding and ionic interactions with Gln266 and Arg221, whereas the hydrophobic furanocoumarin core engages a lipophilic pocket. Most derivatives also showed selectivity for PTP1B over TCPTP, except compound **H17** (Table 3) [86]. These findings support hybrid binding modes combining catalytic-site anchoring with hydrophobic peripheral engagement.

### 6.2. Multitarget PTP1B Inhibitors

Multitarget compounds have attracted increasing interest in diabetes drug discovery because they can simultaneously modulate complementary pathways involved in glucose homeostasis, insulin sensitivity, and diabetic complications. In this context, PTP1B has been combined with several targets, including α-glucosidase, α-amylase, DPP-4, aldose reductase, and vascular endothelial growth factor receptor 2 (VEGFR-2). Despite the conceptual attractiveness of multitarget drug design, achieving balanced inhibition across multiple targets remains a major medicinal chemistry challenge. High potency against one target is frequently accompanied by reduced activity toward others or by suboptimal physicochemical and pharmacokinetic properties. Consequently, multitarget activity demonstrated in enzymatic assays should not be considered synonymous with therapeutically relevant polypharmacology in vivo.

#### 6.2.1. Dual PTP1B/α-Glucosidase Inhibitors

Dual PTP1B/α-glucosidase inhibitors are particularly attractive because they combine improved insulin sensitivity with reduced postprandial glucose absorption. α-Glucosidase hydrolyzes oligosaccharides into absorbable monosaccharides in the small intestine; thus, its inhibition lowers postprandial glycemia, while PTP1B inhibition enhances insulin signaling. Natural dual PTP1B/α-glucosidase inhibitors reported up to 2021 have been reviewed by Paoli and co-workers [49]. Here, we focus on more recent natural products and synthetic dual inhibitors.

**Figure 21:**
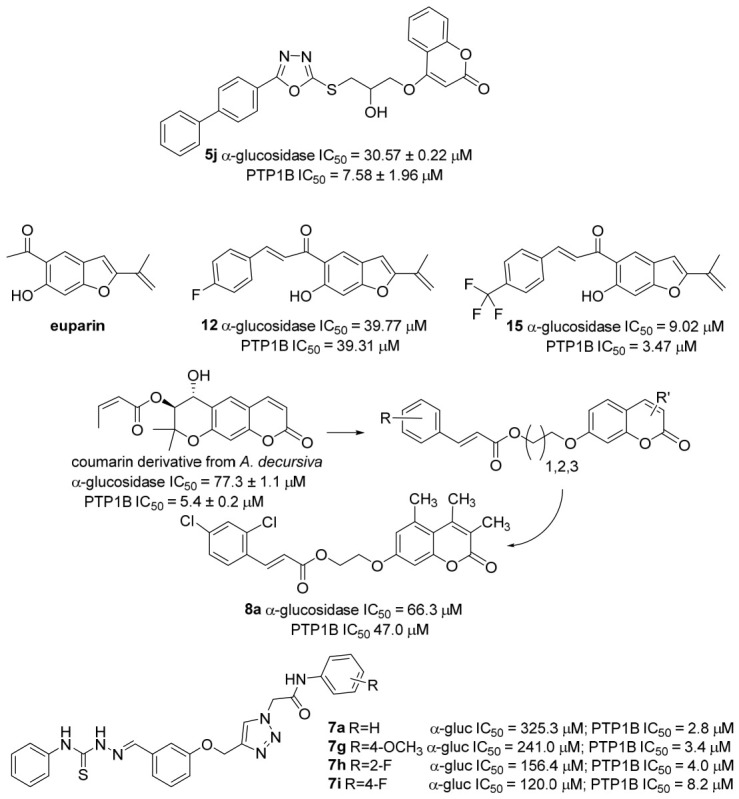
Representative structures of dual PTP1B/α-glucosidase Inhibitors.

Zhao and co-workers synthesized coumarin–oxadiazole derivatives and evaluated them against both enzymes: two series were developed with coumarin substitution at either the 7- or 4-position, with 4-substituted derivatives showing superior activity. Compound **5j** showed an IC_50_ value of 30.57 ± 0.22 µM against α-glucosidase and 7.58 ± 1.96 µM against PTP1B (Figure 21) [87].

Wang and co-workers designed benzofuran-chalcone hybrids inspired by euparin, a natural product from *Eupatorium chinense*. Among these, compound **12** showed IC_50_ values of 39.77 µM and 39.31 µM against α-glucosidase and PTP1B, respectively, whereas compound **15** was more potent, with IC_50_ values of 9.02 µM and 3.47 µM [88]. The improved activity of compound **15** may reflect optimized electronic distribution and spatial orientation of the benzofuran and chalcone units (Figure 21).

Yang and colleagues applied scaffold simplification to coumarin derivatives originally isolated from *Angelica decursiva,* retaining the coumarin core while opening the benzopyran ring and removing chiral centers [89,90]. SAR analysis showed that electron-withdrawing substituents, steric bulk, and linker length influenced α-glucosidase inhibition. Compound **8a** emerged as the most active derivative, with IC_50_ values of 66.3 µM against α-glucosidase and 47.0 µM against PTP1B (Figure 21) [90].

A further hybridization strategy combined a phenoxymethyl-1,2,3-triazole-N-phenylacetamide scaffold with a thiosemicarbazide moiety through a Schiff base linkage. Biological evaluation revealed a markedly different activity profile toward the two targets. While all derivatives showed only moderate α-glucosidase inhibition (IC_50_ = 120–330 µM), a subset of compounds (**7a**, **7g**, **7h**, and **7i**) displayed appreciable PTP1B inhibitory activity, with IC_50_ values ranging from 2.8 to 8.2 µM. (Figure 21) [91]. SAR analysis indicated that α-glucosidase inhibition benefited from electron-withdrawing substituents at the 2- or 4-position, whereas PTP1B inhibition was more tolerant of electronic variation. This divergence highlights the challenge of balancing potency across two mechanistically distinct targets.

**Figure 22:**
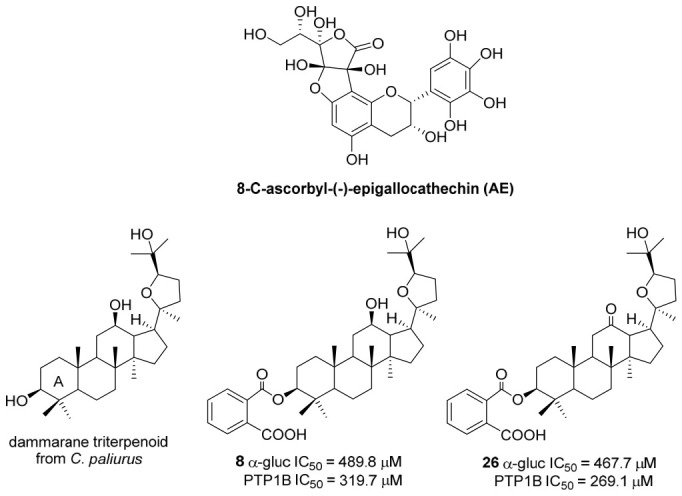
Representative structures of natural multitarget compounds.

Among natural multitarget compounds, 8-*C*-ascorbyl-(−)-epigallocatechin (**AE**), a major polyphenolic constituent of oolong tea, inhibits α-glucosidase with an IC_50_ value of 142.8 µM and has been reported to downregulate PTP1B expression, although no direct enzymatic IC_50_ against PTP1B was provided (Figure 22) [92,93]. Therefore, **AE** may act through complementary mechanisms involving postprandial glucose control and modulation of insulin signaling, but the lack of quantitative PTP1B enzymatic data limits direct comparison with other dual inhibitors.

Dammarane-type triterpenoids from *Cyclocarya paliurus* have also been explored as dual inhibitors [94]. Structural modifications on ring A generated derivatives such as compounds **8** and **26**, which retained dual activity but remained in the high micromolar range (Figure 22). Compound **8** showed IC_50_ values of 489.8 µM against α-glucosidase and 319.7 µM against PTP1B, while compound **26** showed IC_50_ values of 467.7 µM and 269.1 µM, respectively [95]. Although modest in potency, these results support the feasibility of optimizing triterpenoid scaffolds for dual activity.

#### 6.2.2. Dual PTP1B/Aldose Reductase Inhibitors

Dual inhibitors of PTP1B and aldose reductase (AR) represent another strategy for addressing both insulin resistance and diabetic complications. AR, an aldo-keto reductase involved in hyperglycemia-associated complications, shares some pharmacophoric requirements with PTP1B inhibitors, including a polar or acidic head group and a lipophilic aromatic region.

**Figure 23:**
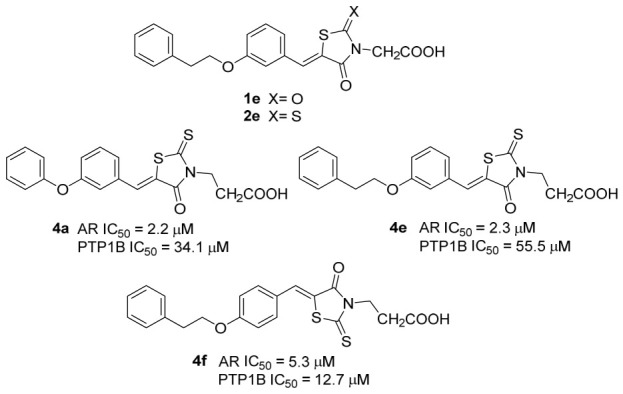
Representative structures of dual PTP1B/Aldose Reductase Inhibitors.

Starting from the 4-thiazolidinedione scaffold, Ottanà and co-workers synthesized derivatives targeting both AR and PTP1B. Compounds **1e** and **2e** were identified as dual inhibitors: both compounds behaved as reversible inhibitors, exhibiting an uncompetitive mode of inhibition against human AR and a non-competitive mechanism against PTP1B. Further optimization led to derivatives, **4a** and **4e**, which showed potent inhibition of human AR, with IC_50_ values of 2.2 and 2.3 µM, but more moderate PTP1B inhibition, with IC_50_ values of 34.1 µM and 55.5 µM. Compound **4f** showed a more balanced profile, with IC_50_ values of 5.3 µM for AR and 12.7 µM for PTP1B (Figure 23) [96]. These results demonstrate the feasibility of exploiting shared pharmacophoric features, although balanced potency remains difficult to achieve.

**Figure 24:**
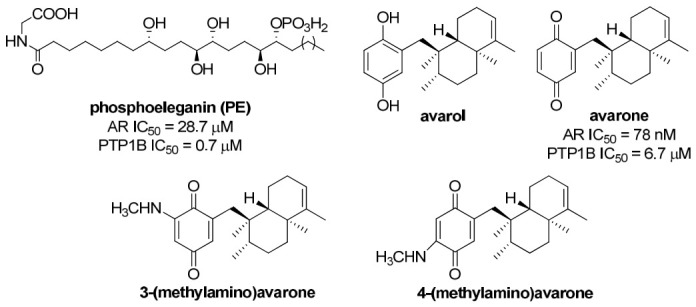
Representative structures of natural dual PTP1B/Aldose Reductase Inhibitors.

Among natural dual AR/PTP1B inhibitors, phosphoeleganin (**PE**), a phosphorylated marine-derived polyketide, acts as a mixed-type inhibitor of AR and a pure non-competitive inhibitor of PTP1B, with IC_50_ values of 28.7 µM and 0.7 µM, respectively (Figure 24) [97]. Its submicromolar potency toward PTP1B and distinct non-competitive mechanism make it a valuable lead for further optimization.

Marine-derived sesquiterpene from *Dysidea avara*, including avarol, avarone, and methylamino avarone derivatives, also showed reversible inhibition of both AR and PTP1B [98] (Figure 24). Avarone was the most potent derivative, with IC_50_ values of 78 nM against AR and 6.7 µM against PTP1B. It showed lower affinity for TCPTP (IC_50_ = 39.2 µM), corresponding to approximately 5.8-fold selectivity for PTP1B. Notably, avarone is a neutral, non-phosphorylated scaffold capable of competitively inhibiting PTP1B, supporting the possibility of designing inhibitors that do not rely on highly charged phosphate-mimetics [99].

#### 6.2.3. Multienzyme Inhibitors Involving PTP1B, α-Glucosidase, α-Amylase and DPP-4

More recently, multitarget design has expanded beyond dual inhibition to include simultaneous modulation of PTP1B, α-glucosidase, α-amylase, and DPP-4. α-Amylase and α-glucosidase regulate carbohydrate digestion and postprandial glucose absorption, while DPP-4 controls glucose homeostasis by rapidly degrading the incretin hormones GLP-1 and glucose-dependent insulinotropic polypeptide (GIP). Inhibition of DPP-4 prolongs the biological activity of these incretins, thereby enhancing glucose-dependent insulin secretion, suppressing glucagon release, and improving glycemic control [100]. Consequently, simultaneous inhibition of these enzymes may provide complementary mechanisms for regulating postprandial glucose levels.

**Figure 25:**
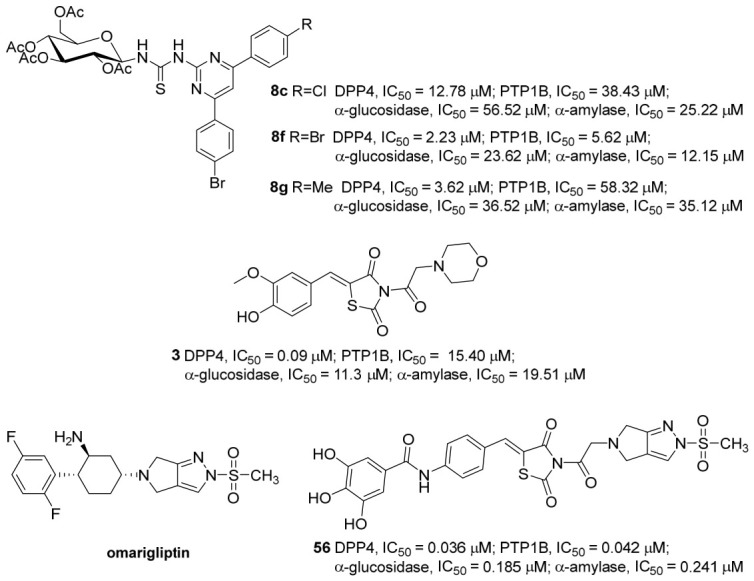
Representative structures of multienzyme inhibitors involving PTP1B, α-Glucosidase, α-Amylase and DPP-4.

Based on known pharmacophoric features of inhibitors targeting these enzymes, pyrimidine-thiourea hybrids functionalized with a D-glucose unit were designed. The pyrimidine scaffold is present in several DPP-4 inhibitors and appears in α-amylase/α-glucosidase inhibitors, whereas thiourea motifs have been reported in dual α-glucosidase/PTP1B inhibitors [101]. Among the synthesized derivatives, compounds **8c**, **8f**, and **8g**, exhibited the most favorable multitarget profiles, with compound **8f** showing the best overall balance of activity (IC_50_ = 12.15 μM against α-amylase, 23.62 μM against α-glucosidase, 2.23 μM against DPP-4, and 5.62 μM against PTP1B (Figure 25) [101].

Rashid and co-workers further developed multitarget inhibitors starting from a vanillin–morpholine hybrid bearing a TZD scaffold (compound **3**, Figure 25), which had previously shown excellent DPP-4 inhibitory activity, together with moderate inhibition of PTP1B, α-glucosidase and α-amylase. Through molecular hybridization with gliptin pharmacophores, several hybrids were generated. Among the new molecules, derivative **56**, carrying the structural motif of omarigliptin, showed the most balanced multitarget profile [102]. This approach illustrates how established DPP-4 inhibitor pharmacophores can be integrated with scaffolds associated with α-glucosidase, α-amylase, and PTP1B inhibition to generate multifunctional antidiabetic candidates.

#### 6.2.4. Rationale for Dual PTP1B/VEGFR-2 Targeting

Given the role of VEGFR-2 in diabetic vascular complications, including diabetic retinopathy and microvascular dysfunction [103,104], simultaneous modulation of PTP1B and VEGFR-2 has emerged as an attractive multitarget strategy. While PTP1B inhibition may improve insulin sensitivity and metabolic control, VEGFR-2 inhibition could counteract aberrant angiogenesis and endothelial dysfunction associated with chronic hyperglycemia. From a pharmacological perspective, the combined modulation of these two targets may provide complementary benefits by restoring insulin signaling while limiting vascular damage [16,105]. However, to the best of our knowledge, no genuine dual PTP1B/VEGFR-2 inhibitors have yet been reported in the literature. Current efforts are mainly directed toward multitarget compounds capable of modulating PTP1B together with additional diabetes-related targets, while the development of single molecules specifically designed to inhibit both PTP1B and VEGFR-2 simultaneously remains an unexplored area of medicinal chemistry.

## 7. Critical Perspective on Dual and Multitarget PTP1B Inhibitors

The development of dual and multitarget inhibitors centered on PTP1B reflects the increasing recognition that T2DM is a multifactorial disorder requiring modulation of interconnected metabolic pathways. Combinations such as PTP1B/α-glucosidase, PTP1B/AR, and more complex assemblies involving α-amylase or DPP-4 aim to improve insulin sensitivity, reduce postprandial glucose excursions, and mitigate long-term complications. From a drug discovery standpoint, these strategies are conceptually attractive but structurally demanding. The electronic and steric requirements of the different targets often diverge, making it difficult to achieve balanced potency within a single molecular framework. As a result, many compounds display asymmetric activity profiles, with preferential inhibition of one target. Furthermore, increasing structural complexity to accommodate multiple pharmacophores may compromise physicochemical properties, permeability, and metabolic stability.

Natural products and marine-derived scaffolds demonstrate that non-phosphorylated and structurally unconventional frameworks can provide meaningful dual activity, sometimes with improved selectivity. However, their translation into drug-like candidates requires careful optimization of potency, selectivity, and pharmacokinetic properties.

Overall, while dual and multitarget PTP1B inhibitors represent an attractive strategy for integrated metabolic control, their successful development depends on achieving balanced activity, coherent structural integration, and favorable drug-like properties. Future progress will likely depend on structure-based multitarget design and early integration of ADME and safety considerations into the discovery process (Table 4). Representative inhibitors discussed throughout this review are comparatively summarized in Table 5 according to their origin, potency, mechanism of action, and selectivity. This overview highlights the major advantages and limitations of the different medicinal chemistry strategies developed to target PTP1B.

The comparison reported in Table 4 highlights the trade-offs between potency, selectivity, and drug-like properties. While catalytic-site inhibitors achieve high potency, allosteric and multitarget approaches offer improved selectivity and pharmacological relevance, supporting a shift toward more integrative drug design strategies. Overall, the evolution from catalytic-site inhibition to allosteric and multitarget strategies reflects a paradigm shift in PTP1B drug discovery, driven by the need to overcome limitations in selectivity and drug-like properties.

## 8. Clinical Trials in Diabetes

**Figure 26:**
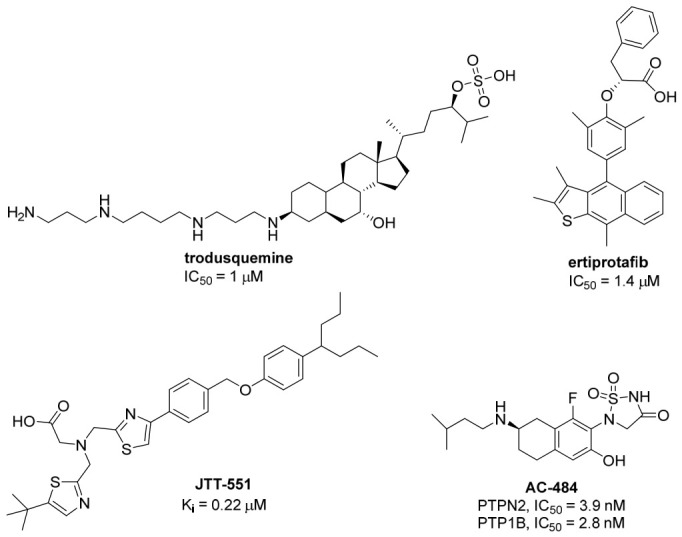
Representative structures of clinically evaluated PTP1B-targeted agents for diabetes and beyond diabetes.

Despite extensive preclinical research, only a limited number of PTP1B-targeted agents have advanced to clinical evaluation. Ertiprotafib [106], trodusquemine [107], JTT-551 [108], and ISIS-113715 [109] progressed to Phase II clinical evaluation; however, none completed clinical development. As discussed below, each program was discontinued or redirected for distinct reasons, including aggregation-mediated off-target effects, insufficient clinical benefit, pharmacokinetic or pharmacodynamic limitations, or trial-related factors such as poor patient enrollment (Figure 26). The advantages and limitations of clinically relevant compounds are summarized in Table 6.

Trodusquemine exhibits approximately 200-fold selectivity over TCPTP, with an IC_50_ value of ~1 µM, and acts as a non-competitive allosteric inhibitor [107]. Mechanistically, trodusquemine binds to the C-terminal domain of PTP1B, thereby confirming its allosteric mode of action [107]. More recently, trodusquemine has attracted renewed interest for the treatment of Parkinson’s disease due to its protective effects against α-synuclein toxicity and TAR DNA-binding protein 43 (TDP-43) proteinopathies [110]. Trodusquemine remains one of the most extensively investigated PTP1B inhibitors because of its unique allosteric mechanism and high selectivity over TCPTP. Nevertheless, despite encouraging preclinical results in obesity and metabolic disorders, its clinical development for diabetes did not progress beyond Phase II. This outcome underscores the difficulty of translating robust enzymatic inhibition into sustained clinical efficacy, even for highly selective allosteric inhibitors.

Ertiprotafib, with an IC_50_ = 1.4 µM, induces aggregation of PTP1B through interaction with its C-terminal region in a unique and specific manner. Although ertiprotafib demonstrated potent PTP1B inhibition in vitro, its clinical development was discontinued because its mechanism of action was later shown to involve enzyme aggregation rather than selective catalytic inhibition. This unusual mechanism, together with off-target effects and an unfavorable pharmacological profile, limited its therapeutic potential and highlighted the importance of achieving selective target engagement through well-defined binding interactions rather than nonspecific protein aggregation. Notably, this mechanism contrasts with that of trodusquemine, which binds to the C-terminal domain without inducing aggregation [111].

JTT-551 acts as a competitive inhibitor, with a K_i_ value of 0.22 µM toward PTP1B and a 42-fold selectivity over TCPTP [108]. JTT-551 represented one of the most potent catalytic-site inhibitors to enter clinical development. However, despite favorable in vitro potency and selectivity, its clinical efficacy proved insufficient to justify further development. This finding further illustrates that high enzymatic potency alone does not necessarily translate into meaningful therapeutic benefit, particularly when pharmacokinetic and pharmacodynamic factors limit target engagement in vivo.

ISIS-113715, an antisense oligonucleotide targeting PTP1B, advanced to Phase II clinical evaluation [112]; however, further development was discontinued due to poor patient enrollment (NCT00330200). Unlike small-molecule inhibitors, ISIS-113715 employed an antisense strategy to reduce PTP1B expression rather than directly inhibiting its catalytic activity. Although this approach offered an alternative mechanism for modulating PTP1B signaling, clinical development was discontinued because of poor patient enrollment, preventing a definitive assessment of its therapeutic efficacy.

Collectively, these clinical experiences indicate that the major challenges in PTP1B drug development extend beyond enzyme inhibition itself. While potent inhibitors have been successfully generated, clinical translation has been limited by issues including suboptimal pharmacokinetics, insufficient target engagement, off-target effects, unconventional mechanisms of action, and trial-related limitations. These findings emphasize that future PTP1B inhibitors must achieve an appropriate balance between potency, selectivity, drug-like properties, and in vivo efficacy.

### PTP1B Inhibitors Beyond Diabetes

More recently, AC484, a dual inhibitor of protein tyrosine phosphatase non-receptor type 2 (PTPN2) and PTP1B, has entered Phase I clinical trials as monotherapy and in combination with programmed death-1 (PD-1)–targeted therapies (NCT04777994, NCT06188975) (Figure 26) [113]. PTPN2 belongs to the PTP family and plays a key role in tumor immunology. Another dual inhibitor developed by Calico Life Sciences and AbbVie, ABBV-CLS-579, whose chemical structure has not been disclosed, is currently undergoing Phase I clinical evaluation as monotherapy or in combination with PD-1 inhibitors for the treatment of locally advanced or metastatic tumors (NCT04417465). Importantly, studies conducted in PTPN2/PTP1B double-knockout mice resulted in embryonic lethality or bone marrow hypoplasia. These findings suggest that simultaneous co-regulation of PTPN2 and PTP1B may not necessarily be beneficial and could lead to unpredictable adverse effects [113].

## 9. Conclusions

PTP1B has long been recognized as an attractive yet intrinsically challenging therapeutic target. From a medicinal chemistry perspective, the main limitation arises from the structural features of its catalytic pocket, which is highly conserved, strongly polar, and optimized for binding negatively charged pTyr residues. These characteristics have historically required the use of highly acidic phosphate mimetics, often resulting in poor membrane permeability, suboptimal pharmacokinetics, and limited selectivity over closely related phosphatases such as TCPTP. The gap between potent enzymatic inhibition and successful clinical translation reflects these fundamental physicochemical constraints.

Clinical experience with early PTP1B inhibitors, including ertiprotafib, trodusquemine, JTT-551, and antisense approaches, demonstrated that pharmacological modulation of the target is feasible. However, safety concerns, adverse effects, and inadequate pharmacokinetic profiles limited their development. These outcomes highlight that, for PTP1B, potency must be carefully balanced with selectivity, drug-like properties, and tissue-specific activity.

Recent medicinal chemistry efforts have shifted away from exclusive catalytic-site inhibition toward alternative strategies. Allosteric modulators, multisite ligands targeting peripheral binding regions, and hybrid molecules inspired by natural scaffolds represent attractive approaches to overcome the limitations of the conserved active site. In parallel, multitarget strategies integrating PTP1B inhibition with modulation of α-glucosidase, α-amylase, DPP-4, aldose reductase, or VEGFR-2 reflect the multifactorial nature of type 2 diabetes and its complications. While polypharmacology offers potential synergistic benefits, achieving balanced potency across multiple targets without compromising safety remains a major challenge.

Advances in structural biology, improved understanding of conformational dynamics, particularly WPD-loop movements and allosteric communication pathways, and progress in computational modeling are reshaping the design paradigm. Future developments will likely depend on the integration of structure-based design, molecular dynamics simulations, and early ADME optimization. In addition, emerging approaches such as reversible covalent modulation, targeted protein degradation, and tissue-selective delivery systems may provide new opportunities to expand the therapeutic windows.

In conclusion, although the path toward clinically successful PTP1B inhibitors remains complex, the evolution of medicinal chemistry strategies over the past decade provides renewed optimism. Although PTP1B remains an attractive target, its clinical translation for diabetes therapy has not yet been achieved. Future progress will depend on improving selectivity, pharmacokinetic properties, and target validation through integrated medicinal chemistry and translational approaches.

## Figures and Tables

**Table 1:** 3-Aryl-5-arylidene-2-thioxo-4-imidazolidinone derivatives.

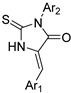	**Compound**	**Ar_1_**	**Ar_2_**	**IC_50_ (µM)**
	**7a**	C_6_H_4_-4-F	C_6_H_4_-4-OC_6_H_5_	3.5 ± 0.3
	**7c**	C_6_H_4_-4-SCH_3_	C_6_H_4_-4-OC_6_H_5_	3.5 ± 0.2
	**7e**	C_6_H_5_	C_6_H_4_-4-C_6_H_5_	2.9 ± 0.2
	**7f**	C_6_H_5_	C_6_H_4_-3-OC_6_H_5_	2.7 ± 0.24

**Table 2:** Chromone and TZD-Based Hybrids.

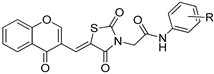		
Compound	R	IC_50_ (µM)
**9**	*p*-CH_3_	1.40 ± 0.04
**10**	*p*-OCH_3_	1.61 ± 0.09
**13**	*p*-NO_2_	16.83 ± 0.54
**17**	*m*-CH_3_	3.48 ± 0.13
**18**	*m*-OCH_3_	2.02 ± 0.10
**21**	*m*-NO_2_	1.50 ± 0.05
**25**	*o*-CH_3_	2.00 ± 0.10
**26**	*o*-OCH_3_	1.67 ± 0.06
**29**	*o*-NO_2_	1.90 ± 0.07

**Table 3:** Selectivity for PTP1B over TCPTP of furanocoumarin derivatives.

Percentage Inhibition at 10 µM		
Compound	TCPTP	PTP1B
**H1**	18.1	90.3
**H6**	11.4	84.2
**H7**	25.9	96.1
**H8**	<10	80.8
**H9**	25.9	96.8
**H10**	12.0	88.1
**H17**	73.5	87.1
**H20**	<10	66.7
**H22**	27.7	90.8

**Table 4:** Comparison of PTP1B inhibitor strategies: natural, synthetic, and multitarget approaches.

Strategy	Advantages	Limitations	Typical Potency	Selectivity	Drug-Likeness	Key Examples
Natural Products	High structural diversity; intrinsic bioactivity; Often associated with favorable biological profiles; ability to bind allosteric/peripheral sites	Moderate potency; complex structures; variability; poor PK optimization	µM (sometimes nM, e.g., EGCG)	Moderate–good	Often limited (polarity, metabolism)	EGCG, coumarins, diterpenes, dammarane triterpenoids, serratane triterpenoids avarone
Synthetic (Catalytic-site)	High potency; well-defined SAR; strong target engagement	Poor selectivity (TCPTP); high polarity; low permeability; poor PK	nM–low µM	Low–moderate	Often poor	JTT-551, phosphate mimetics
Synthetic (Allosteric)	Improved selectivity; better physicochemical properties; non-competitive mechanism	Moderate potency; complex binding; fewer validated sites	µM	High	Improved vs. catalytic	Trodusquemine, imidazolidinones
Multisite (Bidentate)	Enhanced affinity and selectivity; dual binding interactions	Increased molecular weight; synthetic complexity	low µM	Moderate–high	Variable	TZD hybrids, chromones
Multitarget (Dual/Polypharmacology)	Potential for complementary pharmacological actions; addresses multifactorial disease; improved efficacy potential	Difficult SAR optimization; unbalanced activity; risk of off-target effects	µM	Variable	Often reduced (complexity)	PTP1B/α-glucosidase, PTP1B/AR, PTP1B/DPP-4
Natural-inspired Hybrids	Combine diversity + optimization; improved potency vs. natural	Still moderate PK; design complexity	µM–low µM	Moderate	Improved vs. natural	chalcone hybrids, coumarin derivatives

**Table 5:** Representative PTP1B inhibitors: comparison of potency, mechanism, and origin.

Class	Compound	Origin	Target(s)	IC_50_/ K_i_ (PTP1B)	Mechanism	Selectivity (vs. TCPTP)	Key Features
Allosteric	Bergapten	Natural (coumarin)	PTP1B	6.64 µM	Non-competitive	Moderate	Binds allosteric site
Allosteric	Imperatorin	Natural (coumarin)	PTP1B	9.94 µM	Non-competitive	Moderate	Lipophilic coumarin scaffold
Allosteric	Glycyrrhetinic acid Derivatives **4f**	Natural (tritepenes)	PTP1B	2.5 µM	Non-competitive	n.r.	Fused indole scaffold
Mixed	EGCG	Natural (polyphenol)	PTP1B/ LMW-PTP	K_i_ = 26 nM	Mixed-type	Low	Multisite binding; poor drug-likeness
Mixed	Dammarane triterpenoids **7**–**10**	Natural (triterpenes)	PTP1B	4.3–8.6 µM	Competitive/ mixed	n.r.	Triterpenes scaffold
Competitive	Pimarane diterpene (**6**)	Natural	PTP1B	0.33 µM	Competitive	n.r.	Hydrophobic terpene scaffold
Competitive	Serratane triterpenoids **1**	Natural (triterpenes)	PTP1B	6.27 µM	Competitive	n.r.	Triterpenes scaffold
Catalytic + hydrophobic	Furanocoumarin derivatives	Natural-inspired	PTP1B	µM range	Mixed	Good	Dual-site binding (acidic + hydrophobic)
Hybrid	TZD–thiadiazole (**M17**)	Synthetic	PTP1B	0.41–4.68 µM	Non-competitive	n.r.	Multisite interaction
Hybrid	Chromone derivative (**9**)	Synthetic	PTP1B	1.40 µM	Likely mixed	~19-fold	Tunable SAR (EDG/EWG effects)
CADD-derived	IZD-Imidazolidine-2,4-dione (**10**)	Synthetic	PTP1B	2.07 µM	n.r.	n.r.	Virtual screening hit
Dual	Coumarin– oxadiazole (**5j**)	Synthetic	PTP1B/α-glucosidase	7.58 µM	n.r.	n.r.	Multitarget design
Dual	Chalcone– benzofuran (**15**)	Synthetic	PTP1B/α-glucosidase	3.47 µM	n.r.	n.r.	Hybrid natural- inspired scaffold
Dual	Avarone	Natural (marine)	PTP1B/AR	6.7 µM	Competitive	~5.8-fold	Neutral scaffold (non-phosphate)
Dual	PE	Natural (marine)	PTP1B/AR	0.7 µM	Non-competitive	n.r.	Potent natural lead

A comparative analysis of representative PTP1B inhibitors highlights key trends in medicinal chemistry strategies. Catalytic-site inhibitors generally achieve high potency but suffer from limited selectivity and suboptimal drug-like properties due to their reliance on highly polar pTyr mimetics. In contrast, allosteric inhibitors and natural products often exhibit improved selectivity and physicochemical profiles, albeit with moderate potency. Hybrid and multitarget compounds represent an emerging compromise, aiming to balance affinity, selectivity, and pharmacological breadth, although achieving optimal activity across multiple targets remains a significant challenge. n.r. (not reported); IC_5__0_, half-maximal inhibitory concentration; Kᵢ_i_, inhibition constant.

**Table 6:** Clinical Development of PTP1B Inhibitors for Diabetes.

Class	Compound	Origin	Target(s)	IC_5__0_/ Kᵢ (PTP1B)	Mechanism	Selectivity (vs. TCPTP)	Key Features	Clinical Status
Allosteric	Trodusquemine	Synthetic	PTP1B	~1 µM	Non-competitive (allosteric)	~200-fold	Binds C-terminal/ allosteric site; stabilizes open WPD loop	Phase I/II completed
Active-site	JTT-551	Synthetic	PTP1B	Ki = 0.22 µM	Competitive	42-fold	Classical catalytic-site inhibitor	Development discontinued
Aggregation-based	Ertiprotafib	Synthetic	PTP1B	1.4 µM	Aggregation mechanism	Low	Induces oligomerization of PTP1B	Development discontinued
Antisense	ISIS-113715	Synthetic	PTP1B mRNA	n.r.	mRNA silencing	n.r.	Antisense	Phase II terminated (poor enrollment)

Comparison of PTP1B inhibitors in clinical trials in terms of potency, selectivity, mechanism of action, and key characteristics; n.r. not reported.

## Data Availability

No new data were created or analyzed in this study. Data sharing is not applicable to this article.

## Abbreviations

The following abbreviations are used in this manuscript:

T2DM	Type 2 diabetes mellitus
PTP1B	Protein tyrosine phosphatase 1B
IDF	International Diabetes Federation
DPP-4	Dipeptidyl peptidase-4
FFAR1	Free fatty acid receptor 1
PPAR	Peroxisome proliferator-activated receptor
SGLT2	Sodium-glucose cotransporter 2
GLP-1	Glucagon-like peptide-1
PTPN1	Protein tyrosine phosphatase non-receptor type 1
PTPN2	Protein tyrosine phosphatase non-receptor type 2
ER	Endoplasmic reticulum
PTP	Protein tyrosine phosphatase
TCPTP	T-cell protein tyrosine phosphatase
LMW-PTP	Low molecular weight protein tyrosine phosphatase
SPAA	Sulfonylphenylacetamide
SAR	Structure activity relationship
ACP1	Acid phosphatase 1
EGCG	Epigallocatechin-3-gallate
ECG	Epicatechin gallate
TZD	Thiazolidin-2,4-dione
IZD	Imidazolidine-2,4-dione
CADD	Computer-aided drug design
WPD	Trp-Pro-Asp
VEGFR-2	Vascular endothelial growth factor receptor 2
AE	8-C-ascorbyl-(-)-epigallocatechin
AR	Aldose reductase
PE	Phosphoeleganin
TDP-43	TAR DNA-binding protein 43
PD-1	Programmed death-1
pTyr	phosphotyrosine
EWG	Electron-withdrawing group
EDG	Electron-donating group
ADME	Absorption, distribution, metabolism, and excretion
GIP	Glucose-dependent insulinotropic polypeptide
IGF-1R	Insulin-like growth factor-1 receptor
JAK2	Janus kinase 2
PI3K	Phosphatidylinositol 3-kinase
Akt	Protein kinase B
IRS	Insulin receptor substrate
IGF-1	Insulin-like growth factor 1
STAT3	Signal transducer and activator of transcription 3
GLUT4	Glucose transporter type 4
GA	Glycyrrhetinic acid
IF1	Low molecular weight protein tyrosine phosphatase isoform 1
IF2	Low molecular weight protein tyrosine phosphatase isoform 2
IC_50_	Half maximal inhibition concentration
Ki	Inhibition constant
